# Liposome-encapsulated diacyl glycerol and inositol triphosphate-induced delayed oocyte activation and poor development of parthenotes

**DOI:** 10.4274/jtgga.2017.0014

**Published:** 2017-09-01

**Authors:** Ramya Nair, Jyothsna Manikkath, Aswathi R. Hegde, Srinivas Mutalik, Guruprasad Kalthur, Satish Kumar Adiga

**Affiliations:** 1 Department of Clinical Embryology, Central Research Lab, Kasturba Medical College, Manipal University, Manipal, India; 2 Department of Pharmaceutics, Manipal College of Pharmaceutical Sciences, Manipal University, Manipal, India

**Keywords:** Oocyte activation, diacyl glycerol, inositol triphosphate, liposomes, embryo development

## Abstract

**Objective:**

To explore the ability of diacyl glycerol (DAG) and inositol triphosphate (IP3), two major secondary messengers in the calcium signaling pathway, in activating oocytes.

**Material and Methods:**

Oocyte cumulus complex obtained from superovulated Swiss albino mice were incubated in M16 medium with liposome-encapsulated 1,2-Dipalmitoyl-sn-glycerol (LEDAG) and/or IP3 for 3 h. Strontium chloride was used as positive control. The activation potential, ploidy status, and blastocyst rate was calculated.

**Results:**

Both DAG and IP3, individually, induced activation in ~98% of oocytes, which was significantly higher (p<0.01) than activation induced by strontium chloride (60%). Delayed pronucleus formation and a higher percentage of diploid parthenotes was observed in oocytes activated with LEDAG and/or IP3. However, these embryos failed to progress beyond the 6-8–cell stage. Only when the medium was supplemented with LEDAG (5 μg/mL) and IP3 (10 μg/mL) could activated oocytes progress till the blastocyst stage (5.26%), which was lower than the blastocyst rate in the positive controls (13.91%).

**Conclusion:**

The results of the present study indicate that DAG and IP3 can induce delayed oocyte activation and poor development of parthenotes *in vitro*.

## INTRODUCTION

Fertilization is a complex process which involves a series of well-defined morphologic and biochemical events in both spermatozoa and oocytes (1). The entry of sperm into the oocyte initiates a signaling cascade, which hydrolyzes the membrane-bound phosphoinositidyl 4,5 bis phosphate (PIP2) to inositol triphosphate (IP3) and diacyl glycerol (DAG). DAG causes the activation of protein kinase C (PKC) (2), and IP3 binds to IP3 receptors present on the surface of the endoplasmic reticulum (ER) to release Ca^2+^ into the cytoplasm (3), which initiates multiple downstream events required for zygote formation.

The development of an embryo without the involvement of spermatozoa (paternal factors) is known as parthenogenesis. Parthenogenetic embryos play an important role in understanding the paternal and maternal contribution to the development of embryos, act as an alternative source of normally fertilized embryos for quality control experiments in assisted reproductive technology (ART) laboratories, and as a source of embryonic stem cells in the field of regenerative medicine. Even though artificial oocyte activation can be achieved *in vitro* using various physical, electrical, and chemical means (4, 5, 6), *in vitro* development of parthenotes is characterized by increased embryonic arrest, a high degree of fragmentation, developmental delay of embryos (7, 8), decreased blastocyst rate, and low cell number in blastocyst.

The oocyte activation process and the subsequent development of parthenotes is driven by the calcium signaling pathway (7, 9). Artificial oocyte activation agents generate calcium spikes of lower amplitude, which last for a short duration when compared with spermatozoa-induced calcium waves (10). The present investigation was aimed to study whether using DAG and IP3 exogenously as activating agents could improve the developmental potential of activated oocytes. An earlier study showed that microinjection of the secondary messengers in the calcium signaling pathway (DAG and IP3) evoked a calcium spike that was more physiologic and similar to that of natural stimuli (11). However, whether it would be sufficient to drive complete activation of oocytes and support embryo development has not yet been reported. Previous studies used microinjection for the delivery of the secondary messengers for oocyte activation, which requires technical expertise and a sophisticated instrument. In the present study, we used liposome-encapsulated DAG and IP3 individually and in combination to study their oocyte activation potential and the subsequent development of activated oocytes under *in vitro* conditions.

## MATERIAL AND METHODS

### Animal handling

Inbred Swiss albino female mice (8-10 weeks) maintained in the Central Animal Research Facility, Kasturba Medical College, Manipal University, under standard conditions of temperature (25±2 ºC), humidity (45-55%) and light (12:12 h of light and dark) were used for the study. The authors assert that all procedures contributing to this work complied with the ethical standards of the relevant national and institutional guides on the care and use of laboratory animals. Prior approval to carry out the experiment was obtained from the Institutional Animal Ethics Committee of Kasturba Medical College, Manipal (IAEC/KMC/64/2013).

### Preparation of liposomes encapsulated with 1,2-Dipalmitoyl-sn-glycerol

1,2-Dipalmitoyl-sn-glycerol (5 mg, Catalogue no. D9135, Sigma) and soy phosphatidyl choline (45 mg) were dissolved in 15 mL of chloroform in a round-bottomed flask connected to a rotary flash evaporator (ROTAVAP, Buchhi, Switzerland), and chloroform was removed under reduced pressure at 40 °C. A thin film of the lipid mixture and 1,2-Dipalmitoyl-sn-glycerol was obtained after dessication on the inner wall of the flask. Ten milliliters of Milli Q water was added to the flask and agitated using a magnetic bead for 30 min to obtain a milky white liposomal dispersion. The preparation was frozen at -80 °C, lyophilized for 36 h, and the product formed (white powder) was stored in the dark at 4 °C till further use.

### Parthenogenetic activation

Oocyte cumulus complexes (OCC) were obtained by superovulating 8-10–week-old inbred Swiss albino female mice by intraperitoneal (i.p.) administration of 5 IU pregnant mare’s serum gonadotropin (PMSG; Catalogue no. G4877; Sigma, St Louis, MO, USA) followed by 10 IU human chorionic gonadotropin (hCG; Ovutrig-HP, VHB Life Science Inc. GenBiotech, Mumbai, India) 48 h later. The OCCs were collected from the oviduct at 13 h after hCG injection in M2 medium. The oocyte activation was carried out by incubating the OCCs in M16 medium with 10 mM strontium chloride (SrCl_2_; Catalogue no; 107865, Merck) in varying concentrations of liposome-encapsulated diacyl glycerol (LEDAG, 1, 5, 10, 50, 100, 150, 200, 500 µg/mL) and varying concentrations of inositol triphosphate (IP3, Catalogue no. I7012; Sigma, 5, 10, 25, 50, 100, 200 µg/mL) at 37 °C and 5% CO_2_ for 3 h. SrCl_2_ served as a positive control. After 3 h incubation with the activating agents, OCCs were denuded by brief incubation (1 min) in hyaluronidase solution (1 mg/mL in M2 medium) and observed under a phase contrast microscope to check for oocyte activation. Activated oocytes with a single pronucleus and two polar bodies (1PN/2PB) were considered as haploid parthenotes, and those with two pronuclei and one polar body (2PN/1PB) were scored as diploid parthenotes. Parthenotes were washed and cultured *in vitro* in M16 medium to assess their developmental potential and blastocyst rate.

### Statistical analysis

The data pertaining to the activation potential and developmental potential of embryos were represented as percentage and the validity of the data was analysed using the Chi square test using GraphPad InStat 3.0 statistical package (GraphPad Inc, USA). Differences were considered to be statistically significant if p<0.05.

## RESULTS

### Activation potential of liposome encapsulated Dipalmitoyl-sn-glycerol and inositol triphosphate

**Figure 1:**
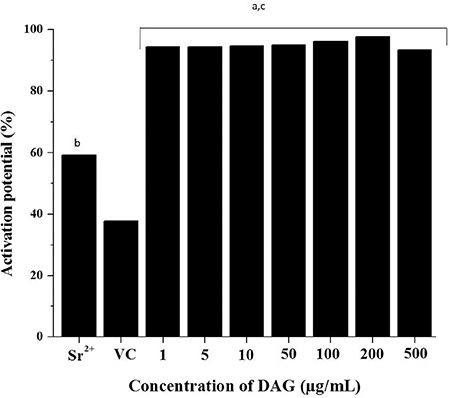
Effect of varying concentrations of diacyl glycerol on activation potential of mouse oocytes. Bar represents percentage of oocytes forming pronuclei with activation. *^a^p<0.001 Sr^2+^ vs. diacyl glycerol all concentrations; ^b^p<0.0001 VC vs. diacyl glycerol all concentrations; ^c^p<0.05 VC vs. Sr^2+^ (n=50, 37, 18, 18, 92, 59, 77, 29, 81, 89 for strontium chloride, vehicle control, 1, 5, 10, 50, 100, 150, 200, 500 µg/mL respectively)*

**Liposome encapsulated Dipalmitoyl-sn-glycerol (LEDAG):** Strontium chloride (positive control) induced activation in 60% of oocytes, whereas liposome alone (vehicle control) induced activation in 40% of oocytes. At all concentrations of LEDAG used in the study (1, 5, 10, 50, 100, 200 and 500 µg/mL), the activation rate was ~98% (Figure 1). However, unlike in the oocytes activated with SrCl_2_, where pronuclei were observed as early as 3 h after exposure, it took at least 48 h for pronucleus formation in the LEDAG group, indicating delayed activation. Incubating oocytes *in vitro* without any activating agents for up to 48 h did not cause any oocyte activation, suggesting that the activation observed in the LEDAG group was not due to ageing of oocytes under *in vitro* conditions.

**Figure 2:**
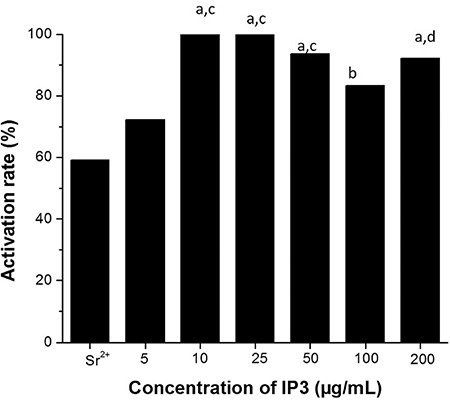
Effect of varying concentrations of inositol triphosphate on activation potential of mouse oocytes. Bar represents percentage of oocytes with pronuclei post activation *^a^p<0.0001, ^b^p<0.001 vs. Sr^2+^; ^c^p<0.0001, ^d^p<0.001 vs. 5 µg/mL of IP3 (n=50, 79, 77, 28, 67, 59, 20 for strontium chloride, 5, 10, 25, 50, 100, 200 µg/mL respectively)*

**Inositol triphosphate (IP3):** The lowest concentration of IP3 (5 µg/mL) used in the study resulted in 75% activation, whereas at 10 and 25 µg/mL concentration, 100% activation was observed (Figure 2). With further increase in IP3 concentrations (50, 100, 200 µg/mL), though the activation rate was marginally reduced, it was significantly higher (p<0.01) than in the positive control, and lower concentrations of IP3 (25 µg/mL). As observed with LEDAG, exposure to IP3 also resulted in delayed pronucleus formation.

**Figure 3:**
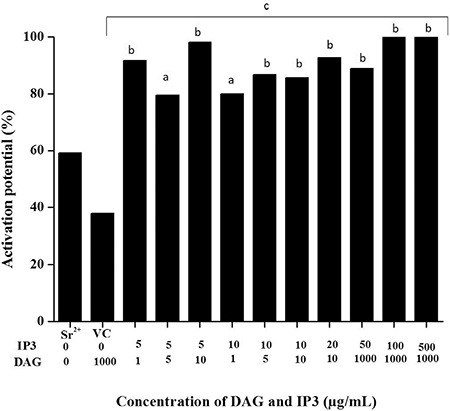
Effect of varying concentrations of inositol triphosphate on activation potential of mouse oocytes *^a^p<0.01 vs. Sr^2+^; ^b^p<0.0001 vs. strontium chloride; ^c^p<0.0001 vs. vehicle control*

**Combination of LEDAG and IP3:** Varying combinations of IP3 and LEDAG were used to test whether combinations of these agents reduced the time required for pronucleus formation. However, LEDAG and IP3 when used in combination, despite having no dose-dependent effect on activation, ~80-90% of oocytes were activated in all different combinations used (Figure 3).

The maximum activation of oocytes was observed in 50 µg/mL of LEDAG and 10 µg/mL of IP3; therefore, only these concentrations were used to check the ploidy status and degeneration/ fragmentation rate of the oocytes.

**Degeneration and fragmentation rate of oocytes activated with LEDAG and IP3:** The degeneration and fragmentation rates were found as 5.13% and 9.2%, respectively, in the positive control group (Sr_2_Cl) (Table 1). Neither in the vehicle control group nor in LEDAG or IP3 group were there any degenerated oocytes. However, 4.47% of oocytes underwent fragmentation in the vehicle control group, similar to the LEDAG (5.15%) and IP3 (5.26%) groups, which was lower than in the positive control group.

**Ploidy status of oocytes activated with LEDAG and IP3:** Oocytes from the positive control group had clear pronuclei formation at 3 h after incubation with SrCl_2_. Parthenotes derived from strontium chloride are usually haploid in nature, which was confirmed from the results of the present study (100% haploid parthenotes) (Table 1). However, both in the LEDAG and IP3 groups, pronucleus formation was observed only at 48 h after incubation, indicating a delayed activation process. In addition, a significantly higher percentage of diploid parthenotes were observed in the LEDAG (76.92%) and IP3 group (43.75%) compared with strontium (p < 0.001). Liposomes alone resulted in the formation of diploid parthenotes in ~ 4% of oocytes.

### Developmental potential of oocytes activated with LEDAG and IP3

**LEDAG:** In the SrCl_2_ group, within 24 h of activation, 92.92% of parthenotes progressed to the 2-cell stage (Table 2). The 2-cell rate in the LEDAG group was in the range of 57-85% and had no concentration-dependent effect (63.16, 84.21, 66.67, 62.67, 82.43, 62.03, and 57.83% in 1, 5, 10, 50, 100, 150, 200 and 500 µg/mL, respectively). Even in the vehicle control group, 60% of parthenotes progressed to the 2-cell stage, indicating that the phospholipids present in soy lecithin or cholesterol itself could induce parthenogenesis. Except in lower concentrations of LEDAG (1 and 5 µg/mL), none of the embryos progressed beyond the 6-8–cell stage. Even in these two concentrations, the embryos were arrested at the morula stage (5.26 and 13.16% in 1 and 5 µg/mL, respectively). This indicates that the use DAG alone is inefficient at driving the whole process of embryogenesis in activated oocytes.

**IP3:** A dose-dependent increase in the 2-cell rate was observed in groups activated with IP3 till 50 µg/mL above which a decrease in the 2 cell rate was observed (80 and 85% in 100 and 200 µg/mL, respectively) (Table 3). The embryos in lower concentrations (10 and 20 µg/mL) progressed till 6-8 cell stage (42.31 and 22.22%), whereas others were arrested at the 4-cell stage. Oocytes activated with IP3 failed to progress beyond 6-8 cell stage at all the concentration used, suggesting that even though the presence of IP3 in activation medium can induce high oocyte activation (delayed), they cannot support the development of early embryos.

**Combination of LEDAG and IP3:** Various concentrations of DAG (1, 5, 10 µg/mL) and IP3 (1, 5, 10, 20 µg/mL) were assessed at different combinations to see whether the activation and developmental potential of parthenotes improved in presence of the two secondary messengers together. In oocytes activated with SrCl_2_, around 23% of parthenotes progressed to the 6-8–cell stage, with a blastocyst rate of 13.91% (Table 4). Oocytes activated with LEDAG alone developed only till the 4-cell stage, whereas 50% of oocytes activated with IP3 developed till the 6-8–cell stage. Except in activation medium containing 10 µg/mL IP3 and 5 µg/mL of LEDAG, the embryos failed to progress to the blastocyst stage in all other combinations. In this group, the blastocyst rate was found to be 5.26%, which was lower than in the positive control.

## DISCUSSION

In this study, we explored the ability of major secondary messengers in calcium signaling pathway, diacyl glycerol and inositol triphosphate as oocyte activating agents. In previous studies, the microinjection approach was used to deliver secondary messengers for oocyte activation (12, 13), which itself is limited by the presence of Ca^2+^ in the injection medium (14, 15), as well as pressure of injection (16). In the present study, IP3 was directly dissolved in the activation medium, and liposomes were used for the delivery of DAG due to their insoluble nature in aqueous solutions.

The use of DAG and IP3, alone and in combination, efficiently activated oocytes, with a lower percentage of degeneration and fragmentation in activated oocytes. Earlier studies observed that microinjection of DAG and IP3 into oocytes could elicit a similar pattern of calcium oscillation as observed during normal fertilization (2, 11, 12, 13). The presence of liposomes could induce activation in oocytes indicates that phospholipids present in soy lecithin and cholesterol also have the potential to cause artificial oocyte activation. To support this, earlier reports available in the literature showed that cholesterol could cause oocyte activation by increasing the intracellular calcium concentration (17), and phospholipids could cause oocyte activation through activation of protein kinase C (18).

One of the major limitations of using these agents for oocyte activation was the delay observed in the process of pronucleus formation, which took almost 48 h, unlike in strontium chloride, which takes only 3 h. Strontium chloride is known to cause long-lasting Ca^2+^ transients during artificial oocyte activation (13). In contrast, the microinjection of IP3 and its agonist was reported to result in calcium oscillations in a higher frequency than during normal fertilization (9, 13, 19, 20, 21), leading to a rapid desensitization of calcium stores (19, 20).

Strontium-induced oocyte activation is mediated through TRPM ion channels activating store-operated calcium (SOC) entry to refill endoplasmic stores to generate the transient calcium wave (22). Even though DAG activates TRPM channels directly (23, 24, 25), it fails to activate its downstream signaling molecules (24, 26) and SOC (23). Even uptake of Sr^2+^ and Ca^2+^ was hindered by the addition of DAG due to inactive SOC (23). A similar result was also observed in our study; the addition of Sr^2+^ after DAG activation failed to rescue delayed activation caused by DAG (data not shown). We hypothesize that the Ca^2+^ entry during DAG induced oocyte activation is through activating plasma membrane SOCs (23), which results in slow Ca^2+^ entry to oocytes and hence the delayed activation process. In the present study, we used 1,2-Dipalmitoyl-sn-glycerol, which has saturated fatty acids. It may be interesting to see whether the type of fatty acid present in DAG has any significant difference in calcium metabolism because wide varieties of saturated and unsaturated fatty acids are found in the cell membrane of the oocyte. Similarly, it is difficult to explain why IP3 causes a similar delay in activation and poor embryo development. It could be due to the shorter half-life of IP3 (27) and therefore, may require frequent additions of IP3 to induce the sufficient calcium spike required for the development of parthenotes. In addition, whether the soluble IP3 present in the medium can efficiently enter the oocyte must be ascertained in further experiments.

**Figure 4:**
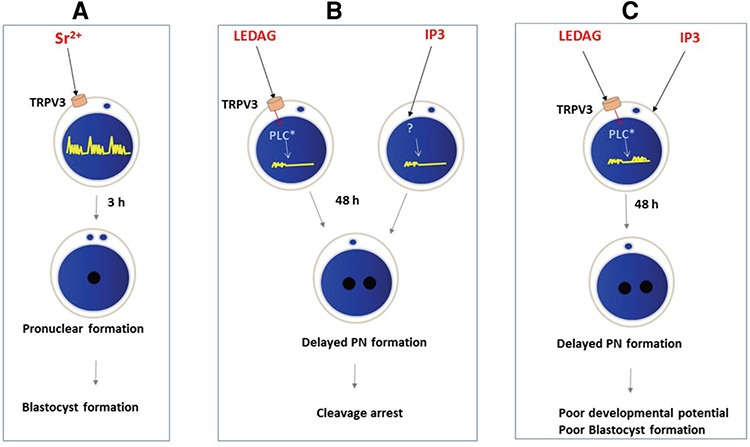
Mode of action of diacyl glycerol and inositol triphosphate in oocyte activation. A) Mode of action of strontium chloride-induced oocyte activation as proposed by Carvacho et al. (22). B) and C) Expected mode of action of diacyl glycerol and inositol triphosphate alone and in combination in inducing oocyte activation LEDAG: Liposome-encapsulated diacyl glycerol; Sr^2+^: Strontium; PN: Pronucleus; PLC*: Inactivated phospho lipase C; TRPV3: Transient receptor potential cation channel, subfamily V, member 3

DAG and IP3, individually, were not capable of driving the development of parthenotes till the blastocyst stage. A similar observation was made by Schoenbeck et al. (11), who found that microinjection of DAG (1,2-Dioctanoyl-sn-glycerol) activated porcine oocytes efficiently but resulted in an arrest at the 4-cell to 6-8–cell stage. The poor development observed in the present study could also be related to the increased oocyte ageing because pronucleus formation itself takes almost 48 h from the collection of OCCs. Aged oocytes are known to have poor developmental potential *in vitro* (28, 29). However, when both these agents are used together, the intracellular calcium rise may be much higher than when they are used alone. The proposed mode of action of DAG and IP3 in oocyte activation is depicted in Figure 4.

During artificial activation of oocyte, cytochalasin D, a microtubule inhibitor, is usually used to derive the diploid parthenogenetic embryos (30, 31). Both DAG and IP3 were shown to increase the percentage of diploid parthenotes obtained after activation. In mouse oocytes, the use of protein kinase C as artificial oocyte activating agents are reported to form embryos without the extrusion of the polar body (2, 32, 33). Earlier studies proved that DAG and IP3 could activate protein kinase C (34, 35). Therefore, use of DAG and IP3, alone or in combination, can help to derive diploid parthenotes without using any microtubule inhibitors. The toxicity exerted by microtubule inhibitors on the development of embryos (36, 37) can be minimized because they are part of the calcium signaling pathway. This is further supported by the low degeneration rate and fragmentation of activated oocytes observed in our study.

In conclusion, liposomes can be used as an approach to deliver DAG across the oolemma. DAG and IP3, when used individually or in combination, induce delayed oocyte activation and poor embryo development. These agents may have potential application in developing diploid parthenotes when used along with efficient activating agents such as strontium because they are shown to give rise to 50% of diploid parthenotes without using any cytokinesis inhibitors during activation. Further studies are essential to understand the exact mechanism of action of these two agents in inducing delayed oocyte activation when supplemented exogenously.

## Figures and Tables

**Table 1:**
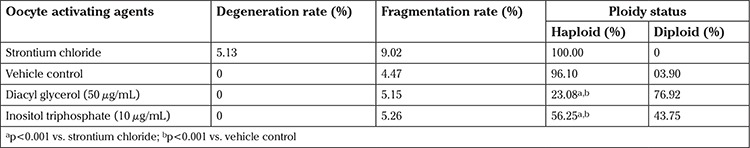
Degeneration and fragmentation rate of oocyte after activation with various agents

**Table 2:**
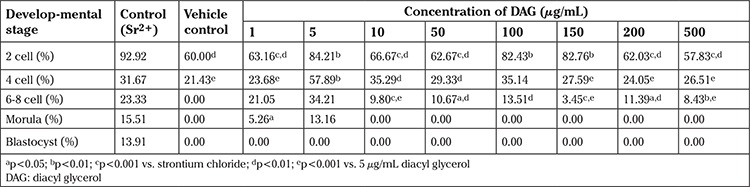
Developmental potential of oocytes activated with 1,2-Dipalmitoyl-sn-glycerol

**Table 3:**
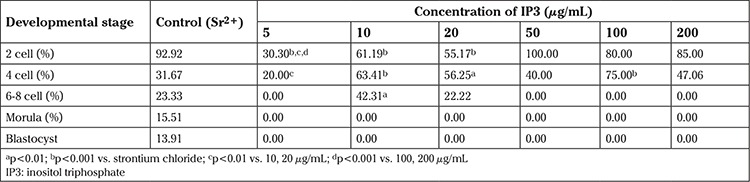
Developmental potential of oocytes activated with inositol triphosphate

**Table 4:**
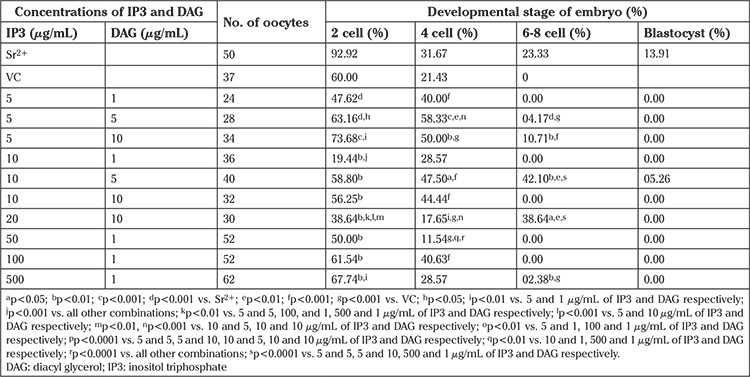
Developmental potential of oocytes activated with varying combinations of inositol triphosphate and diacyl glycerol
